# The Impact of Urbanization on Urban Heat Island: Predictive Approach Using Google Earth Engine and CA-Markov Modelling (2005–2050) of Tianjin City, China

**DOI:** 10.3390/ijerph20032642

**Published:** 2023-02-01

**Authors:** Nadeem Ullah, Muhammad Amir Siddique, Mengyue Ding, Sara Grigoryan, Irshad Ahmad Khan, Zhihao Kang, Shangen Tsou, Tianlin Zhang, Yike Hu, Yazhuo Zhang

**Affiliations:** 1 School of Architecture, Tianjin University, Tianjin 300272, China; 2 Centre for Research in Agricultural Genomics (CRAG) CSIC-IRTA-UAB-UB, Campus UAB, Bellaterra, 08193 Barcelona, Spain; 3 School of Civil Engineering, Tianjin University, Tianjin 300272, China

**Keywords:** land surface temperature, urban heat island, land use cover change, urban planning and development, ecological evaluation, urban system

## Abstract

Urbanization has adverse environmental effects, such as rising surface temperatures. This study analyzes the relationship between the urban heat island (UHI) intensity and Tianjin city’s land cover characteristics. The land use cover change (LUCC) effects on the green areas and the land surface temperature (LST) were also studied. The land cover characteristics were divided into five categories: a built-up area, an agricultural area, a bare area, a forest, and water. The LST was calculated using the thermal bands of spatial images taken from 2005 to 2020. The increase in the built-up area was mainly caused by the agricultural area decreasing by 11.90%. The average land surface temperature of the study area increased from 23.50 to 36.51 °C, and the region moved to a high temperature that the built-up area’s temperature increased by 1.5%. Still, the increase in vegetation cover was negative. From 2020 to 2050, the land surface temperature is expected to increase by 9.5 °C. The high-temperature areas moved into an aerial distribution, and the direction of urbanization determined their path. Urban heat island mitigation is best achieved through forests and water, and managers of urban areas should avoid developing bare land since they may suffer from degradation. The increase in the land surface temperature caused by the land cover change proves that the site is becoming more urbanized. The findings of this study provide valuable information on the various aspects of urbanization in Tianjin and other regions. In addition, future research should look into the public health issues associated with rapid urbanization.

## 1. Introduction

The rapid growth of urban areas worldwide has been observed over the past few decades [1]. The main factors contributing to urbanization are the lack of economic development and the increasing population [2]. Despite the slow growth of the global population, it is still expected that the number of people will continue to increase by around 2030 [1]. According to estimates, the world’s urban area is expected to grow by over a million kilometers by 2030 [1,3,4,5,6]. Urbanization is most prevalent in developing countries due to rapid economic development. China is one of the most prominent in the world regarding urbanization. It has been estimated that the country’s urban land area expanded at an annual rate of 13.3% [7].

Urbanization positively impacts people’s lives, as it allows them to improve their living standards and reduce their energy consumption. It can also help mitigate climate change by reducing vehicle miles travelled and greenhouse gas emissions [8]. Unfortunately, there are still negative impacts of urbanization. Due to the human activities that have occurred in the past few decades, the city has expanded. This process has caused both positive and negative effects [9].

Urbanization is a complex process involving multiple modelling variables and mechanisms involved in its development. The various aspects of this process must be thoroughly studied to understand its effects. One of the most effective ways to predict an urban area’s characteristics is through Land Use Cover change (LUCC) analysis [8,10,11]. A comprehensive simulation of the urban development process is necessary in today’s world [12,13]. With the help of spatial data, such as land area and development characteristics, urban models can be used to study the patterns of urbanization. These models can also simulate the conditions affecting the city’s development. Urban models use mathematical equations to describe the urban system [14,15]. They can also deal with the various factors that affect the development of a city. The study results are based on the interactions between different strategies and aspects [9,16]. Urban models are becoming more effective at predicting future changes in the LUCC due to the complexity of the process. They can use the available data and conditions to model the different factors affecting the city’s development. Numerous studies have been conducted on the use of LUCC in policy formulation and decision making.

However, the application of cellular automata and the Markov process is relatively rare. Tianjin is considered one of the most prominent cities in China that has experienced sustained urbanization, industrialization, and urbanization in China [17,18]. As a result of its ongoing development, many cities are expected to continue to grow [19]. It is essential that the cities’ LUCC change be studied and analyzed to determine its future trend [20,21]. This study was conducted to comprehensively analyze the various factors that have affected the city’s development. The study examined the LUCC change in Tianjin from 1995 to 2015. It first created five maps with different classifications at different points in time. The analysis revealed that many areas were converted into built-up areas. The model was then analyzed to create a set of dynamic variables for the Cellular Automata Model (CA) [11,22,23]. These variables were then used to project the LUCC change in the city from 2025 to 2050.

## 2. Materials and Methods

### 2.1. Study Area

**Figure 1:**
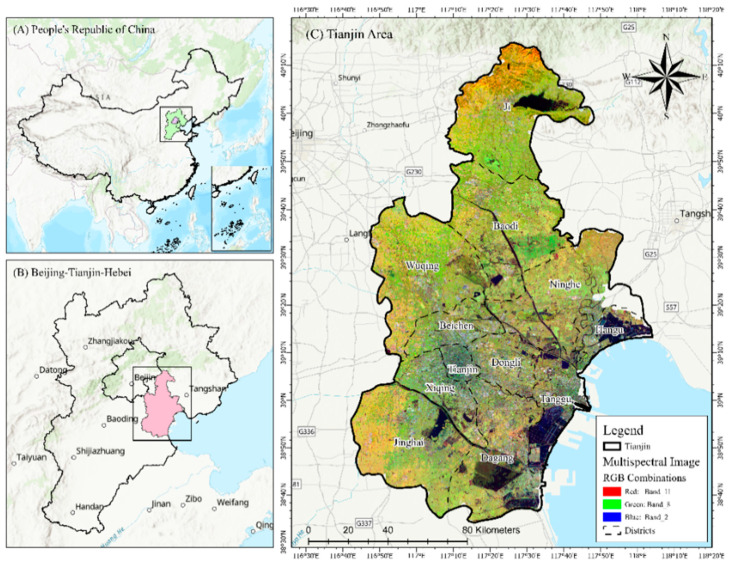
Geographic location and characterization of the study area: (**A**) People’s Republic of China; (**B**) Beijing–Tianjin–Hebei (TBH); and (**C**) multispectral satellite image of Tianjin city.

The city of Tianjin, the largest city on China’s northern coast, is straddled at 38°34′ N to 40°15′ N and 116°43′ E to 118°04′ E (Figure 1), having a thousand square kilometers. It is regarded as the fifth-largest city in the country after Shanghai, Beijing, Guangzhou, and Shenzhen [24]. With a warm, temperate, semi-humid monsoonal climate, it is characterized by four distinct seasons during the year [25,26]. Over the past few years, Tianjin has experienced massive urbanization, with its population increasing from 12.99 million in 2010 to 13.86 million in 2021 [1].The city of Tianjin has a gross domestic product of about 240 billion yuan, making it one of the most prominent economic centers in China’s northern region [25,27,28]. It is an international port city and has experienced rapid urbanization over the past few decades. Due to rapid urbanization, large areas of land, such as forests, farmland, and meadows, have been converted into built-up areas [29,30].

### 2.2. Acquisition of Spatial Dataset

The United States Geological Survey (USGS) provided cloud-free images of the study area, which were taken from path 170 and series 053, through its website (http://earthexplorer.com) [6,9,10,13,17,25,31]. Due to the varying time of day and night in the study area, the data collected by the Landsat 5 Thematic Mapper (TM) and the Landsat 7 Enhanced Thematic Mapper (ETM) were used to create the LUCC map [13,18,32,33,34]. The data collected by the two satellites (Table 1) were also used to calculate the Normalized Difference of Vegetation Index (NDVI) and Land Surface Temperature (LST).

### 2.3. Methodology

**Figure 2:**
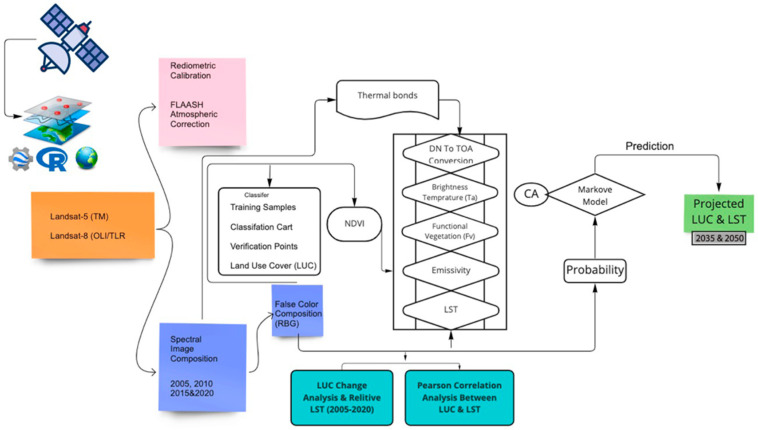
Flowchart of the methodology for the present study.

An integrated workflow template (Figure 2) was used to perform a series of steps. We began by processing the information sets in GEE to create a false colour positive (FCC) [10,11,25,35,36]. A georeferenced map of the outer boundaries of Tianjin was used to extract and mask the study area from all spatial ideas. The support Vector Machine (SVM) classification method was applied to improve the supervised classification results obtained from Landsat imagery [13,30,37,38]. Then, the LST was calculated to determine the time zones in the city [17]. A Pearson correlation analysis was performed based on the land cover, average LST, and percentage of greened and non-greened areas from 2005, 2010, 2015, and 2020 [8,31,39,40]. The CA-Markov model was used to forecast future trends for LUCC and LST in 2035 and 2050 [41]. All spatial statistical analyses and maps were created using ArcGIS 10.7, and ggplot2, corrplot and psych packages used in RStudio [42].

### 2.4. Land Use Cover Change (LUCC) Calculation

**Figure 3:**
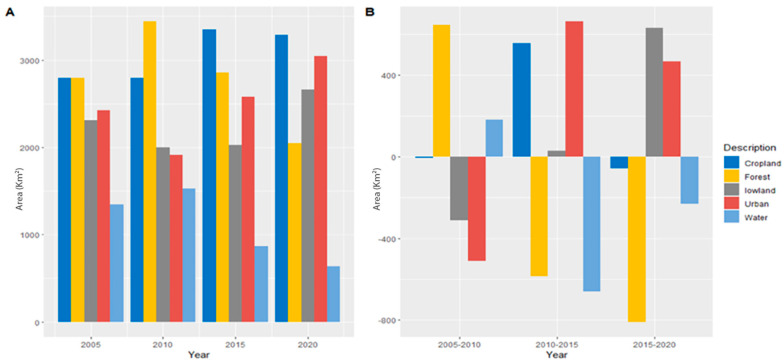
(**A**) Shows the land use land cover area, and (**B**) proportional changes of LUCC during 2005-2020.

Landsat imagery (Landsat-5 TM & Landsat-8 OLI) was used to map the LUCC of Tianjin city for a four-time frame (2005, 2010, 2015, and 2020). The Support Vector Machine (SVM) classification algorithm in GEE was used to classify land use and areas [43,44]. Five types of LUCC were identified: built-up land, cropland, lowland, forest, and water body (Figure 3A). Built-up land included artificial structures such as buildings, roads, and other impervious surfaces. Water included rice fields, reservoirs, and rivers [28,35,45].

At specified intervals, GEE was used to assess the accuracy of the classification results. Field reference points were collected using a Google Earth explorer, which collected field reference average of 250 points for 2005, 2010, 2015, and 2020.

The classification accuracy of the signatures and images was evaluated by creating a confusion matrix consisting of rows and columns that refer to the categories derived from the image. The matrix rows are labelled with the reference values, while the columns represent the categories identified using the same criteria. The total number of entries that formed the main diagonal was then divided by the number of pixels. The Kappa coefficient was calculated using Equations (1)–(3) [1,2,46,47]:

(1) $$P_{0}=\sum_{i=1}^{r}\left(P_{i+}*P_{+i}\right)$$

(2) $$P_{c}=\sum_{i=1}^{r}\left(P_{i+}*P_{+i}\right)$$

(3) $$K^{\Lambda }=\frac{P_{0}-P_{c}}{1-P_{c}}$$

where *r* = the number of rows in the error matrix; *P_ij_* = The proportion of pixels in a row “*i*” and column “*j*”; and *P_i_* = the fraction of the marginal sum of row “*i*”.

### 2.5. Calculation of Land Surface Temperature (LST)

The Landsat-8 thermal infrared sensor (TIRS) of bands 10 and 11 and the OLI sensor of bands 2–5 were used individually to convert the raw image into a radiance spectral image (SR) by following the equations (Table 2) step by step.

### 2.6. CA-Markov Prediction Model Analysis

This model uses a stochastic Markov probability matrix to predict the transition from one state to another [14,43,50]. The study aims to analyze the various effects of urbanization on the land use and development of the city of Tianjin using a computer model known as a Markov chain model. This model was used to predict land use and development trends [13,36,51]. A conditional probability formula was used to estimate trend lines from Equations (4)–(6).

(4) $$S\left(t+1\right)=P_{ij}\times S(t)$$

(5) $$P_{ij}=\left[\begin{array}{ccc} P_{11} & P_{12} & P_{1n}\\ P_{21} & P_{22} & P_{2n}\\ P_{n1} & P_{n2} & P_{n3} \end{array}\right]$$

However,

(6) $$\left(0\leq P_{ij}<1\;and\;,\sum_{j=1}^{N}P_{ij}=1,(i,j=1,2,3\ldots \ldots \ldots .n\right)$$

Because of Markov chain and cellular automata modelling, LUCC and LST’s future scenarios are calculated by projecting 2035 and 2050 using Terrset’s land use change modeler (LCM) (Clark Labs TerrSet 18.31).

## 3. Results

### 3.1. Changes in LUCC between 2005 and 2020

According to the LUCC distribution values for 2005, 2010, 2015, and 2020, the built-up area in cities has increased (Figure 3). Built-up area increased from 15.46% in 2005 to 17.80% in 2010, 19.56% in 2020, and 22.72% in 2050. In 2005, the study area included 18.43% of the lowlands; this number decreased to 12.52% by 2010, 11.89% by 2015, and 10.21% by 2020. Arable land increased rapidly from 26.10% in 2005 to 28.95% in 2020, while other land decreased from 26.47% to 26.10%. Increasing migration from villages to cities has led to an expansion of cultivated land outside prime locations. The cultivated area decreased from 10.72% in 2005 to 7.98% in 2020. Water covered 1.21% of the site in 2005, 0.92% in 2010, 0.87% in 2015, and 0.68% in 2020. An assessment of land use changes during 2005–2020 showed that farmland in the northeastern study area was converted to urban areas (mainly industrial areas). Between 2005 and 2020, built-up urban land and cropland increased by 15.45% and 1.64%, respectively, while lowland land decreased by 13.73%. These results show that about 11.45% of the lowlands have been converted into built-up areas. The LUCC changes were classified into five categories LUC with corresponding definitions (Table 3).

The results of all studies show that urban built-up has changed significantly over two decades. In recent decades, Tianjin has gone from a village to a city residential settlement. This transition happens between agricultural land to residential areas. Urban growth and LST are sensitive to accuracy assessment [29]. According to [52,53], a method was defined for assessing the accuracy of the classification of maps. According to the LUCC maps, the overall accuracy was 84.39% in 2005, 90.43% in 2010, and 94.11% in 2020. Kappa coefficients for the LUCC maps were 0.79, 0.87, and 0.92. The kappa coefficient should be greater than 0.75 or 0.80 to show compatibility between the classification and the reference data [54]. The United States Geological Survey (USGS) recommends using Landsat satellite images for LUCC mapping if the accuracy level is 85% [55]. Our accuracy evaluation results are consistent with those recommended in the literature.

### 3.2. Relationship between LUCC and LST

**Figure 4:**
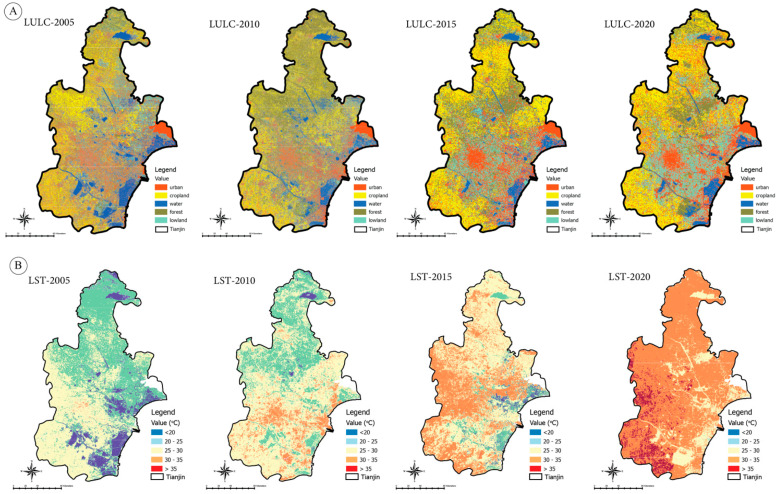
(**A**) Maps for land use land cover (LUCC) classes: (i) water, (ii) vegetation, (iii) forest, (iv) urban, (v) barren land, (vi) cropland; (**B**) land surface temperature (LST) was divided into five thermal categories: (i) <20 °C, (ii) 20–25 °C, (iii) 25–30 °C, (iv) 30–35 °C, and (v) >35 °C of Tianjin between the study period of 2005 to 2020, at 5-year intervals.

**Figure 5:**
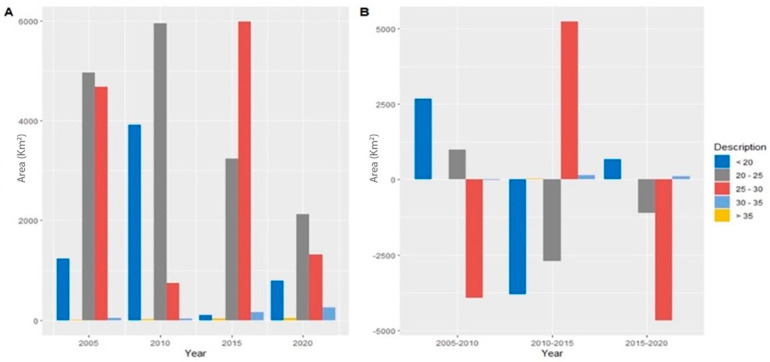
(**A**) Distribution of land surface temperature, and (**B**) proportional changes of LST during 2004–2019.

LST is significantly affected by land use changes (LUCC). The number and distribution of hotspots increase with LUCC types (especially urban expansion) [56]. A map of LST distribution was created using Landsat TM/ETM+/OLI imagery for the study area (Figure 4). There were temperature variations from 21 °C to 43 °C in 2005, 21.8 °C to 44.3 °C in 2010, 22.1 °C to 44.9 °C in 2010, and 22.5 °C to 45.9 °C in 2020. During 2005–2020, built-up urban areas had the highest average temperatures, followed by lowland, cropland, vegetation, and water. In 2005, all LUCC categories had the most elevated average temperatures (Figure 5A). In 2005, urban built-up areas had an average LST of 38.43 °C, 38.99 °C in 2010, 41.86 °C in 2015, and 44.80 °C in 2020. During 2005–2020, the temperature in built-up urban areas LST decreased by 4.12 °C but increased by a maximum of 6.82 °C from 2010 to 2020. Lowlands had the second-highest LST for all LUCC categories during the study period. The LST for wasteland decreased by 3.38 °C from 2005 to 2010 but did not change significantly between 2010 and 2020. The LST for cropland was 31.04 °C in 2015 and increased to 31.98 °C, 32.63 °C, and 33.75 °C in 2010, 2015, and 2020, respectively.

From 2005 to 2020, the temperature of cropland LST decreased by 3.85 °C, while it increased by 15.35% in developed areas. Vegetated areas recorded a decrease of 5.32 °C between 2005 and 2010 LST but an increase of 6.83 °C between 1999 and 2015. All LUCC categories recorded the lowest LST in 2010, and the average temperature in water bodies and vegetated areas was the lowest overall. According to statistics from LST for 2005–2020, the maximum difference between urban areas and water bodies is 14.35 °C.

**Figure 6:**
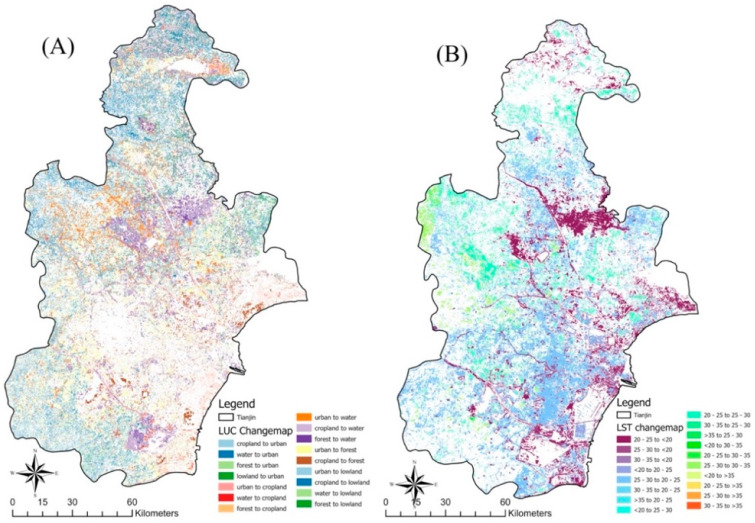
Spatial distribution map for change detection of (**A**) LUCC (km^2^) and (**B**) LST (°C) during 2005–2020.

LST has remained relatively stable between 2005 and 2020. These areas are also referred to as lowlands. Compared to urban areas, lowlands have a higher LST value. Our study came to similar conclusions. High LST values may be found in these areas due to the soil composition (sand, clay, etc.). The average daily air temperature may influence the LST values at the satellite imagery data on the day the satellite imagery was taken rather than the spatial values of the land use classes. To verify that LST results calculated from the Landsat TM/ETM heat band are comparable to actual field temperatures, temperatures of the various LUCC properties must be measured from field observations [57]. Considering the values reported at LST, the daily mean air temperatures of the reported data (the daily mean air temperature on 19 June 2005 is 27.5 °C; on 10 July 2010, it is 23.03 °C; on 23 July 2015, it is 26.86 °C; and the daily mean air temperature on 11 August 2020 is 29.53 °C) are all parallel to each other (See Figure 6).

**Figure 7:**
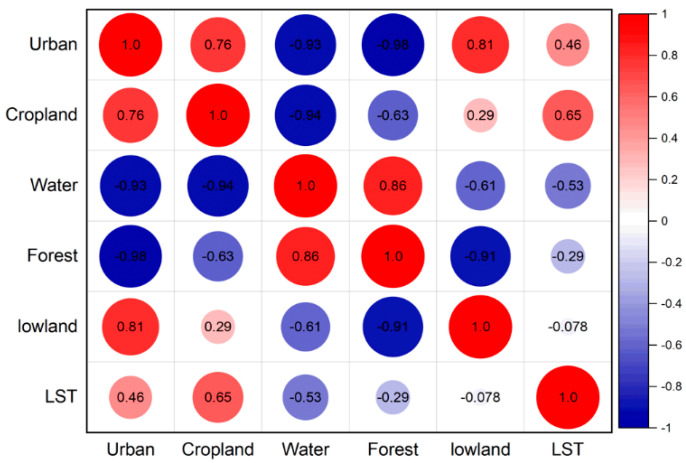
Pearson correlation analysis of land use change and land surface temperature during 2005-2020.

Pearson’s correlation analysis shows LST is statistically associated with populated/developed areas. Even though LST is bad for water and plants and does not have much to do with them, it is strongly linked to forested areas. In the same way, LSTs in cities have a negative and insignificant effect on water and plants. LST and urban/built-up areas have a significant and favourable relationship, as shown by the simple correlation coefficient [51]. In urban areas, a temperature rise may also be caused by the construction of new buildings, highways, businesses, and industrial regions. Negative and insignificant correlations are observed with barren land, while optimistic and negligible correlations are marked with arable and cropland. Pearson correlation analysis results are reported for all LUC variables and LST indices (Figure 7).

### 3.3. Variations of LST Changes over Different LUCC

**Figure 8:**
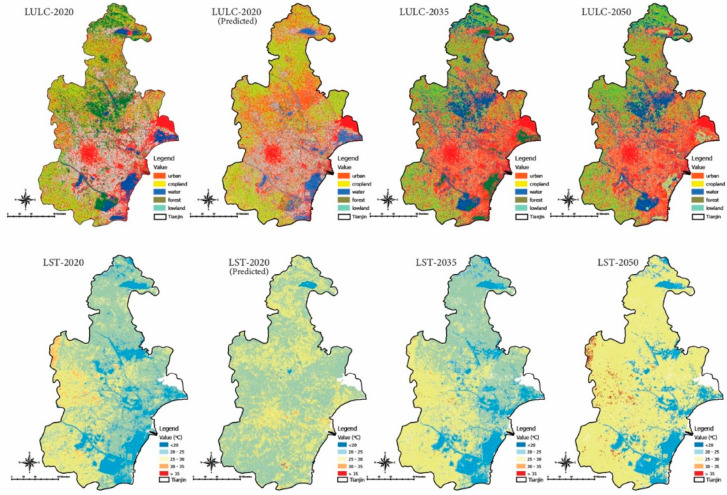
Shows classified maps of LUC and LST for the year 2020, and predicted maps of 2020, 2035, and 2050.

We estimated the mean LST distributions for LUCC classes over 2005–2020. During the study period, mean values of LST increased significantly in all LUCC classes, but matters of LST were substantially higher in built-up areas and bare ground. The importance of LST in the built-up area increased from 28.86 °C to 37.23 °C between 2005 and 2020, while in the empty ground area, they increased from 21.56 °C to 25.01 °C. Over the past two decades, the average LST distribution in built-up and bare-ground regions has risen by about 9 °C and 4 °C, respectively. The LST distribution in water bodies and vegetated areas have also changed. In 2000, the mean LST for vegetated areas was 21.31 °C, but it is expected to reach 25.98 °C by 2020. The LST of water bodies increased from 20 to 24.45 °C. The following figure (Figure 8) briefly describes the changes in LUCC types and their relative impacts on land surface temperature.

### 3.4. Validation of Predicted LUCC and LST Scenarios

To validate the accuracy of the predicted values, we first used the CA-Markov model to estimate the LUCC and LST for 2020 (Figure 8). Based on various kappa parameters, the predicted and estimated maps were compared using the land use Change Modeler in Clark Lab’s Terrset software. The average error value for all parameters during the comparison was about 12.86%, and all kappa parameters, percentage of accuracy, and total kappa values were above 0.80.

### 3.5. Predicted LUCC for 2035 and 2050

We could predict the scenario for 2035 and 2050 based on the classified maps for the study period. According to the predicted LUCC map, the growth of urban areas will be concentrated by 37% in the northwestern and central regions if the trend of the building continues without planned actions. Urban areas will replace the lowlands and vegetation cover. Vegetation cover has decreased by 9.62% from 12.82% in 2020. Based on the study scenario, LUCC would face a 20.51% increase in developed land, followed by a significant decrease in lowlands, vegetation cover, and water bodies of 10.87%, 9.62%, 8.32%, and 2.45%, respectively (Figure 8). The category-wise land use statistics for the forecast years are shown in the following table: Ecosystem services, urban health, and thermal characteristics may be affected by decreased vegetation cover and increased urbanization. If unplanned urban expansion continues, the environmental, economic, and medical problems will increase significantly. A proper land use plan, the protection of water bodies, and the reforestation of forests are needed to make Tianjin city more environmentally sustainable.

By forecasting LST for 2035 and 2050, the simulation showed that higher temperatures will occur in the built-up areas in the northwest and central parts of the country (Figure 8), ranging from 41.56 °C to 44.34 °C in 2035 and 2050, respectively. We divide the temperature zone into five classes to estimate how much area is covered by each temperature range (Table 4). Based on the projections, LST has increased over the past two decades (2005–2020), with urban areas influencing the prevalence of LST. UHI effects will increase as urban areas and vegetation cover decrease. It would be possible to explain the temperature increase without urbanization by climate change, greenhouse effects, and surface features. The LST prediction highlights the real risks of the temperature rise in the trend, including higher UHI effects. A combination of energy use, greenhouse gas emissions, and air pollution contribute to the UHI effect. It threatens aquatic systems (rivers, lakes, ponds, streams, and oceans) and human health. Human health is primarily harmed by increased greenhouse gas emissions, which affect urban health and reduce the urban environment’s sustainability [58].

### 3.6. Limitations of the CA-Markov Model

The prediction of LUCC and LST can be improved using the CA-Markov model if the previous LUCC and LST patterns are consistent. As a result, CA-Markov models do not provide accurate spatial predictions for raster datasets [59]. Since influential factors can be directly determined between CA-Markov and other factors, CA-Markov is based on a probability matrix [60]. Given the relative importance of the different variables in identifying the most important variables, it is essential to note that the CA-Markov model generates training patterns and automatically begins training after receiving inputs from the strata. The input parameters are not individually weighted according to established standards [61]. Since urbanization, the loss of green space and increase in surface temperatures are primarily influenced by human activities and conscious decisions at regional to metropolitan scales; it is impossible to predict them accurately. It is essential to recognize that dynamic models have some limitations. Still, they help develop hypotheses and make decisions about changes in land cover or surface temperatures in any given area, regardless of their rules. In recent years, LUCC and LST variability and predictive maps have emerged as one of the best tools for managing and mitigating vital natural resources.

## 4. Discussion

Tianjin’s rapid urbanization and development between the 1990s and 2020s significantly altered the LUCC landscapes caused by farmland separation and reduced total vegetation cover [25,33]. The city’s urban development also resulted in the establishment of new industries and residential areas. Rapid vegetation cover loss affects an area’s natural cooling effect [29,62]. Some factors contributing to this phenomenon are vegetation shading and transpiration. To amplify this, LST and NDVI have shown that VC, due to its cooling effect, serves as a sink in an urban heat island [11,63]. Rapid vegetation cover loss has several consequences for an area’s natural cooling effect. It has the potential to eventually eliminate the processes that regulate surface transpiration and evaporation [11,17,56,64]. Urbanization leads to distorted construction, reducing soil infiltration and increasing surface runoff. As a result, the water table and groundwater table decrease. Evapotranspiration is not adequately realized due to these two factors. Climate change leads to a deterioration of the water balance [49]. Climate variables such as daily maximum and minimum temperatures are affected by changes in land use. Surface albedo changes due to changes in land use. Therefore, land use changes disturb the balance of Earth’s radiation [65]. An important factor in reducing air temperatures is the conversion of wetlands to agricultural land with high albedo [66].

Although the impact of this phenomenon on the LST of various types of plants is less than that of urban tree cover and gardens, studies have shown that it still contributes to the overall reduction of the area’s natural cooling effect [32,67]. The impact of various types of urban vegetation, water bodies, and forests on the LST varies according to their proportional area [23,37]. In urban areas, vegetation plays a vital role in controlling or mitigating temperature. Evaporation from urban water bodies contributes to moisture accumulation in the surrounding air. According to studies, these bodies regulate the LST in residential areas. It is also known that urban areas contribute to the development of intricate heat flows within these regions [46,68]. Various private and public entities have worked together to revitalize large tracts of land for industrial, commercial, and residential development. Traditional wooden structures have been demolished and replaced with tall structures made of non-evaporative materials such as glass, concrete, and aluminium. These materials can directly impact heat flows in urban areas [8,10]. According to studies, urban areas in China are more vulnerable to severe LST than rural areas. LST has risen due to the government’s decision to convert agricultural and forest land into urban areas [38,69]. The government has relocated factories and businesses to the outskirts of cities to improve their efficiency. These facilities are typically found in developed areas. Before the development of urban areas, forests and vegetation were regarded as buffer zones between rural and urban areas, absorbing excess heat generated by factories and automobiles [3,29,40]. According to the scientific literature, the cooling effect of LUCC is well-matched to the expected warming effect caused by the physical interaction of the Indian region and its surroundings [32]. For example, the maximum cooling contribution from forested areas is 0.27%, while the minimum cooling effect is 0.06%. The most negligible difference between the surface temperature and the impervious surface is the primary reason why vegetation contributes the least to the cooling effect. The greatest cooling effect, on the other hand, is observed when forested areas are converted into water bodies. This is due to the fact that the contribution of land cover to cooling is negligible in various areas, such as urban areas, water bodies, and vegetation. The results of the study revealed that the built-up area in the southeastern and central port areas will continue growing. The paper discussed the various effects of the LUCC on the Tianjin city’s development. The study used the CA model and Geographic Information Systems to analyze the data. The results of the analysis helped improve the Tianjin city’s planning process. In addition, the paper discussed the use of remote sensing tools for improving the urban planning process.

## 5. Conclusions

The objective of this study was to analyze the influence of LUCC on land surface temperature (LST) in a large urban area of Tianjin. Data from RS were used to observe the area’s various socioeconomic and development parameters. The study also used the CA-Markov model and Pearson correlation coefficient to evaluate the contribution of landscape dynamics to temperature. A 5.94% increase in built-up area was found to increase the temperature by 1.5%. However, the increase in vegetation cover by 10% showed a negative correlation. In addition, the study concluded that LUCC has a cooling effect of about 1.40 °C in the city. The average warming effect of LUCC on the UHI is about 0.5%.

On the other hand, the cooling effect of LUCC compared to the shifts in the reverse direction is 0.11%. The positive contribution of LUCC to the UHI was higher than the negative one. Urban development and infrastructure planning should be further targeted to minimize the impacts of climate change. In addition to improving water bodies and parks, other measures, such as the establishment of green spaces and linear planting of woody plants, should also be implemented. The study found that further research is needed to analyze the impact of land use change on the climate of regions and cities. As more areas are affected by climate change, the government and private sector must work together to develop effective cooling strategies. Environmental education should be made accessible to promote the development of ecological resources. This needs effective urban planning and green policies to address the increasing thermal stress. In addition, a quantitative analysis of these parameters needs to be conducted. Although the study found that urbanization directly impacts land surface temperature, it is not yet clear how the effects of this process are related to the other factors. The practical application of the study provides essential guidance for urban landscape planning. It shows how landscape connectivity between impervious and green areas can affect LST. Future research should also address infrastructure stress and public health issues associated with rapid urbanization.

## Figures and Tables

**Table 1:** The Landsat data used in this study are outlined in detail.

Period-Images	Satellite Sensor ID	Spatial Resolution
2005 May–September	Landsat-5 TM	30 m|100 m
2010 May–September	Landsat-5 TM	30 m|100 m
2015 May–September	Landsat-8 OLI_TIRS	30 m|100 m
2020 May–September	Landsat-8 OLI_TIRS	30 m|100 m

**Table 2:** Stepwise process for Land surface temperature (LST) determination.

Steps	Process Name	Equations	References
1	Spectral Radiance (SR)	$L_{\lambda }=0.0003342*DN+0.1$	[16,23,48]
2	Brightness Temperature (T_B_)	$T_{B}=\frac{K_{2}}{\ln\left(\left(K_{1}/L_{\lambda }\right)+\left.1\right)\right.}-273.15$	[11,16,24]
3	NDVI	$NDVI=\left(NIR-RED/NIR+RED\right)$	[11,25,32]
4	Fractional Vegetation (F_v_)	$F_{v}=\left(\frac{NDVI-{NDVI}_{min}}{{NDVI}_{max}-{NDVI}_{min}}\right)$	[16,25,33,38]
5	Surface Emissivity (Sε)	$S\varepsilon =0.004*F_{v}+0.986$	[16,38,40,47]
6	Land Surface Temperature (LST)	$LST=\frac{T_{B}}{1+\left(\lambda_{\sigma }T_{B}/\left(hc\right)\right)\ln\varepsilon }$	[15,38,40,49]

where λ is the effective wavelength (10.9 mm for a thermal band in Landsat 8 data), σ is the Boltz–Mann constant (1.38 × 10^−23^ J/K), h is the Plank constant (6.626 × 10^−34^ Js), and c is the speed of light in vacuum (2.998 × 10^−8^ m/sec).

**Table 3:** Land use cover (LUC) statistics in 2035 and 2050.

Category	LUC_2035		LUC_2050	
	Area	%Age	Area	%Age
urban	3001.75	18%	3204.70	19%
cropland	401.69	2%	272.27	2%
water	1716.04	10%	1678.71	10%
forest	1982.96	12%	1946.75	11%
lowland	9914.57	58%	9914.57	58%

**Table 4:** Land surface temperature (LST) statistics in 2035 and 2050.

Category	LST-2035		LST-2050	
	Area	%Age	Area	%Age
<20 °C	1839.763	16.85%	1851.812	16.96%
20–25 °C	4933.693	45.18%	5056.619	46.31%
25–30 °C	4118.498	37.72%	3859.214	35.34%
30–35 °C	26.86548	0.25%	150.4464	1.38%
>35 °C	0.113955	0.00%	0.842528	0.01%

## Data Availability

On request, the authors will provide the data from this study.
